# Postpartum sepsis-like illness and rash associated with Hansen’s disease

**DOI:** 10.1515/crpm-2021-0046

**Published:** 2022-02-23

**Authors:** Jinai Bharucha, Lynne Saito-Tom

**Affiliations:** Department of Obstetrics, Gynecology, & Women’s Health, John A. Burns School of Medicine, University of Hawai’i, Honolulu, HI, USA

**Keywords:** Hansen’s disease (HD), HD reactions, postpartum, pregnancy, rash in pregnancy

## Abstract

**Objectives:**

Hansen’s disease (HD) often manifests during pregnancy and the postpartum. Patients with HD may experience reactions that mimic other conditions making diagnosis challenging.

**Case presentation:**

We present a case of a patient from Chuuk, a state of the Federated States of Micronesia with a sepsis-like illness and worsening painful rash immediately postpartum. Antepartum, the patient noted a pruritic rash on her legs. Four hours after delivery, the patient became febrile and later developed systemic inflammatory response syndrome (SIRS). The rash rapidly spread to other areas of her body and became painful and edematous. Eight weeks after delivery, a skin biopsy revealed tuberculoid granulomatous dermatitis consistent with HD.

**Conclusions:**

HD and its associated reactions are easily misdiagnosed. Performing a skin biopsy of unusual skin lesions or common skin lesions with severe illness in a pregnant patient can expedite diagnosis of rare conditions such as HD. Early initiation of treatment for HD and its reactions are critical to prevent serious nerve damage and permanent disability.

## Introduction

Hansen’s disease (HD), also known as Leprosy, affects four million people worldwide and is a chronic granulomatous infectious disease caused by *Mycobacterium leprae* [1]. Due to the fear and stigma associated with leprosy, HD is the preferred term in the United States. HD is found primarily in tropical and subtropical regions. In 2015, the WHO reported the highest incidence of HD cases in India, Brazil, and Indonesia (>10,000 cases) and a large number of cases (1,000–9,999 cases) in parts of Africa, South East Asia, and the Philippines [2]. HD is also epidemic in the Federated States of Micronesia and the Republic of the Marshall Islands [3].

Transmission of HD occurs through the upper respiratory tract and skin lesions and requires prolonged exposure to an infected individual. The majority of the population (95%) has an effective innate immune response that is protective against acquiring the disease. The average incubation period is from 9 months to 20 years, making presentation often unexpected [1].

The clinical features of HD depend on the bacterial proliferation, immunologic response of the host, and degree of neural involvement. Initial clinical signs range from a single hypopigmented macule to a more diffuse rash [4]. Skin rashes, its primary presenting symptom, have a broad differential diagnosis in pregnancy. Many rashes of pregnancy are relatively benign and self-resolving, therefore not necessitating dermatology referral. The diagnosis of HD requires a skin biopsy [5].

HD is easily misdiagnosed because of its varied incubation period and clinical features. Its manifestations can be confused with syphilis particularly because of its tendency to result in false positive rapid plasma reagin [6]. In addition, acute hypersensitivity or immunological reactions occur in up to 50% of all patients. These reactions can be confused with cellulitis, drug eruptions, tumid lupus, sepsis, and a systemic connective tissue disease flare [5].

Multidrug therapy for 1–2 years is required for complete treatment of HD. Without treatment, nerve damage will lead to deformity and disability. Although patients may have nerve damage or neuritis at time of diagnosis, prompt treatment is imperative to prevent further damage and sometimes to even reverse damage. Even with treatment, 30–40% of patients will have possible additional nerve damage caused by reactions during treatment. When left untreated for years, disabilities caused by HD are permanent [5]. Therefore, prompt diagnosis is critical.

During pregnancy, alterations in the immune response impair cell-mediated immunity, therefore, pregnant women are more susceptible to manifestations and reactions of HD. HD reactions are managed with prednisone. All medications used to treat HD may affect the fetus, therefore patients may choose to stop treatment during pregnancy or take multidrug therapy at lower dosages with close surveillance during pregnancy [5]. This pregnant woman with a rash on her leg was hospitalized in the intensive care unit postpartum and found to have HD.

## Case presentation

**Figure 1:**
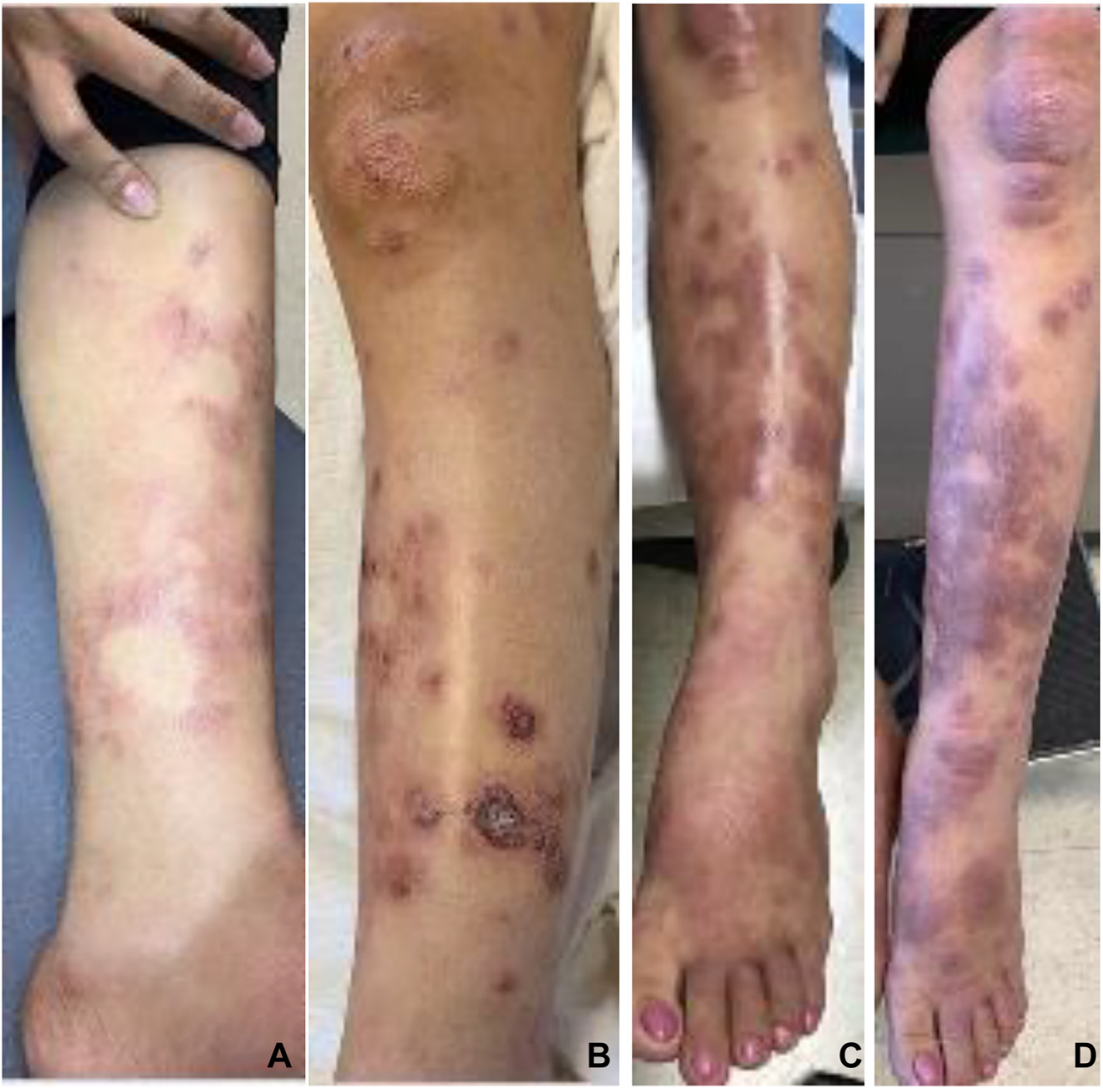
Progression of rash on patient’s left lower extremity during Hansen’s disease reaction. (A) Initial presentation of rash consistent with Hansen’s disease (HD) on left shin at 35 weeks gestation. (B) Three days postpartum: rash on left shin with crusting ulcers. (C) Four weeks postpartum: rash on left shin with lower extremity and pedal edema. (D) Ten weeks postpartum: rash on left shin with lower extremity and pedal edema, prior to starting multidrug therapy.

A 20-year old woman, gravida 2 para 1001, at 35 5/7 days of gestation presented to the outpatient clinic to establish prenatal care and reported a pruritic rash on her legs that appear only during pregnancy. She recently moved to Hawaii from Chuuk, a state of the Federated States of Micronesia, where she had limited prenatal care. The patient denied any past medical history. During her first pregnancy in Chuuk, 18 months prior, she had noted a pruritic rash on her legs that resolved after she had an uncomplicated vaginal delivery with a normal male infant. She denied any family members with a similar rash. The patient’s left leg was noted to have erythematous plaques with a few scabs from ankle to knee and a few hypopigmented patches (Figure 1A).

She was admitted for early labor at 38 4/7 days of gestation and proceeded to have a spontaneous vaginal delivery. Neonatal Apgar scores were 8 and 9 at 1 and 5 min, respectively, with a birth weight of 3,997 g. After placental delivery, intermittent uterine atony was noted requiring administration of methylergonovine maleate and misoprostol with subsequent improvement in uterine tone. The estimated blood loss from the delivery was 450 mL. Within 4 h of delivery, the patient became febrile to 102.4 F with tachycardia, prompting initiation of gentamicin and clindamycin for presumed endometritis. On physical exam, the patient was noted to have a plaque-like rash on her bilateral lower extremities and perineum and small erythematous patches on her bilateral upper extremities.

On postpartum day #1, the patient was hypotensive with worsening, painful rash. Physical exam revealed urticarial wheals throughout chest, arms, buttock, and thighs, and her left lower extremity had crusting lesions with the background rash (Figure 1B). Due to concern for a drug reaction, gentamicin and clindamycin were discontinued. The patient required intensive care admission with a norepinephrine drip and two units of packed red blood cells to stabilize her blood pressure. She was transitioned to vancomycin, metronidazole, and aztreonam for possible infectious etiology. Famotidine and Cetirizine were added for histamine blockade. On postpartum day #4, the patient stabilized off norepinephrine with resolution of urticaria but persistent left lower extremity lesions with central eschars. She was discharged on postpartum day #5 with topical clobetasol and mupirocin.

At her two week postpartum check, the patient reported that the rash was improving with clobetasol and mupirocin. On physical exam, a scattered annular rash was noted over bilateral arms and legs. At her four week postpartum check, she reported the rash was still painful. On physical exam, urticarial papules, plaques, and polycyclic wheals in various stages of healing, some with scale and crusting extending down bilateral legs to plantar surface of feet, worse on left lower extremity with 2+ pitting edema (Figure 1C). She also had polycyclic wheals scattered on her abdomen, back, and bilateral upper extremities with associated erythema. At six weeks postpartum, the patient’s rash continued with worsening pain and increased leg swelling, therefore oral prednisone was started, which caused improvement, but not complete resolution of the rash. The patient was referred to dermatology and at eight weeks postpartum, a skin biopsy of a lesion showed tuberculoid granulomatous dermatitis, consistent with HD.

The patient was referred to the Hansen’s Disease Community program and an infectious disease specialist. Three months postpartum, the patient’s neurologic exam revealed loss of sensation in bilateral feet and retained sensation in bilateral hands. She was started on multidrug therapy for HD and continued on prednisone.

## Discussion

In hindsight, the patient’s antepartum rash and postpartum course were both manifestations of untreated HD. We suspect that the postpartum systemic illness and painful rash were likely a Type 2 Erythema Nodosum Leprosum reaction caused by the depression of the cell-mediated immune system in pregnancy. Other postpartum skin conditions on the differential diagnosis for the patient’s skin rash and systemic inflammatory response syndrome (SIRS) included systemic lupus erythematosus, pemphigoid gestationis, polymorphic eruption of pregnancy, or another autoimmune condition. However they were ruled out by the skin biopsy.

Prompt identification of HD facilitates early treatment and avoids its debilitating lifelong disability. Literature review suggests that HD often manifests during pregnancy and the postpartum period. This was first noted by Ryrie in 1938, who managed a hospital full of HD patients. He noted that out of about 600 women, there was a marked effect of pregnancy on the progression of HD [7]. Later in the 1960s, a retrospective review of 62 pregnancies in 26 treated patients found that onset was related to pregnancy in two patients. Over half (53%) of patients with active lepromatous disease had co-morbidities during pregnancy such as anemia and pre-eclampsia. Nearly one-third (32%) had a reaction characteristic of Erythema Nodosum Leprosum.

The acute hypersensitivity or immunological reaction to *M. leprae* occurs in up to 50% of all HD patients and can occur at anytime during the course of the disease (before, during or after completing multidrug therapy). HD reactions put patients at a higher risk of nerve damage and subsequent disability and deformity. The two main categories of reactions are the Reversal Reaction (Type 1) and Erythema Nodosum Leprosum (Type 2). A third type of reaction called the Lucio phenomenon is rare and occurs in diffuse non-nodular lepromatous leprosy [8].

Type 1, Reversal Reactions are delayed-type hypersensitivity reactions resulting from increased cell-mediated immunity to *M. leprae* antigens [8]. The clinical manifestations of Type 1 reactions include erythema and edema of existing skin lesions, fever, edema of the hands and feet, and occasionally ulceration in severe cases, however systemic symptoms are unusual. Patients may also suffer nerve damage with associated swelling and pain. Reversal Reactions occurs during or even several years after drug treatment is completed but can also be seen in untreated patients with HD.

Type 2, Erythema Nodosum Leprosum reactions are thought to be due to antigen-antibody immune complex formation which elicits an inflammatory response. Type 2 reactions most likely occur during multidrug therapy but can occur at any time. Clinically, Erythema Nodosum Leprosum manifests with rapid appearance of tender, painful transient red nodules that may ulcerate with a predilection for the shins. The skin lesions resolve after 7–10 days followed by mottled hyperpigmentation. The reaction is often accompanied by fever and a clinical presentation resembling SIRS [5]. Postpartum, our patient’s existing rash quickly spread and became more erythematous, edematous and painful, and she developed sepsis.

The key presenting symptom of HD is a rash, which can be hypopigmented patches, erythematous macules, patches, or plaques, nodules, or leonine facies. Pain, loss of sensation, and preferential location of rash on extremities are all distinguishing factors. There may also be anhidrosis, alopecia, recurrent nosebleeds, texture changes of the skin, eye problems, fever, joint pain, or malaise. Our patient presented with hypopigmented patches at her first prenatal visit. The second most likely manifestation of HD is nerve damage which manifests as cranial nerve palsy, corneal damage, nasal depression, or enlarged peripheral nerves. All easily palpable peripheral nerves, such as the great auricular nerve, should be examined for enlargement.

Patients with HD should postpone childbearing until multi-drug therapy is complete. Therapy typically takes 12–24 months and the medications used can be teratogenic. For pregnant patients, treatment of disease and reactions should be continued with modifications to avoid teratogenicity. If HD is recognized during pregnancy, treatment should be initiated promptly to render the bacteria non-communicable and since reactions and neuritis may be exacerbated [5]. The treatment is fully covered by the National Hansen’s Disease Program for all patients. Reactions are typically treated through immunosuppression with corticosteroids to try to avoid permanent tissue and nerve damage [9].

The effects of HD also extend to the fetus; in one review, approximately 20% were premature and 17% ended with fetal demise [6]. In addition, infants born to women with HD have a greater likelihood of low birth weight and growth retardation. Untreated nursing mothers may transmit HD while breastfeeding [4].

HD is a rare condition that can be easily misdiagnosed as evidenced by this case. Its long incubation period and tendency to be exacerbated during pregnancy makes it crucial for all obstetricians to include on their differential diagnosis for rash and systemic illness in pregnant patients. The diagnosis can be easily made with a skin biopsy of a lesion. Many patients are only able to have health insurance during pregnancy and briefly postpartum while on Medicaid. When the patients are uninsured, they may not seek out medical care nor be aware of resources such as the National Hansen’s Disease Program if they develop manifestations of HD. Therefore, it is crucial to diagnose HD when it presents in pregnancy because it may lead to long term morbidity. Particularly in patients with history of travel to endemic areas, HD should be included in the differential diagnosis for all pregnant patients presenting with a rash particularly when accompanied by loss of sensation or other systemic symptoms resembling an HD reaction.

## Take-home message

– Prompt identification and treatment of HD, which often presents for the first time in pregnancy, facilitates early treatment and avoids its debilitating lifelong disability.
– Pregnant patients are particularly susceptible to acute HD reactions which can be confused with cellulitis, drug eruptions, sepsis, and a systemic connective tissue disease flare.
